# Biomechanical walking mechanisms underlying the metabolic reduction caused by an autonomous exoskeleton

**DOI:** 10.1186/s12984-016-0111-3

**Published:** 2016-01-28

**Authors:** Luke M. Mooney, Hugh M. Herr

**Affiliations:** Center for Extreme Bionics, MIT Media Lab, Massachusetts Institute of Technology, Cambridge, MA USA

**Keywords:** Exoskeleton, Biomechanics, Metabolic Power, Walking

## Abstract

**Background:**

Ankle exoskeletons can now reduce the metabolic cost of walking in humans without leg disability, but the biomechanical mechanisms that underlie this augmentation are not fully understood. In this study, we analyze the energetics and lower limb mechanics of human study participants walking with and without an active autonomous ankle exoskeleton previously shown to reduce the metabolic cost of walking.

**Methods:**

We measured the metabolic, kinetic and kinematic effects of wearing a battery powered bilateral ankle exoskeleton. Six participants walked on a level treadmill at 1.4 m/s under three conditions: exoskeleton not worn, exoskeleton worn in a powered-on state, and exoskeleton worn in a powered-off state. Metabolic rates were measured with a portable pulmonary gas exchange unit, body marker positions with a motion capture system, and ground reaction forces with a force-plate instrumented treadmill. Inverse dynamics were then used to estimate ankle, knee and hip torques and mechanical powers.

**Results:**

The active ankle exoskeleton provided a mean positive power of 0.105 ± 0.008 W/kg per leg during the push-off region of stance phase. The net metabolic cost of walking with the active exoskeleton (3.28 ± 0.10 W/kg) was an 11 ± 4 % (*p* = 0.019) reduction compared to the cost of walking without the exoskeleton (3.71 ± 0.14 W/kg). Wearing the ankle exoskeleton significantly reduced the mean positive power of the ankle joint by 0.033 ± 0.006 W/kg (*p* = 0.007), the knee joint by 0.042 ± 0.015 W/kg (*p* = 0.020), and the hip joint by 0.034 ± 0.009 W/kg (*p* = 0.006).

**Conclusions:**

This study shows that the ankle exoskeleton does not exclusively reduce positive mechanical power at the ankle joint, but also mitigates positive power at the knee and hip. Furthermore, the active ankle exoskeleton did not simply replace biological ankle function in walking, but rather augmented the total (biological + exoskeletal) ankle moment and power. This study underscores the need for comprehensive models of human-exoskeleton interaction and global optimization methods for the discovery of new control strategies that optimize the physiological impact of leg exoskeletons.

## Background

Only recently have exoskeletons been shown to reduce the metabolic cost of human locomotion [1–4]. Reducing the metabolic cost of legged locomotion is considered one of the most important roles of a lower-extremity exoskeleton [5, 6]. Even if reducing metabolic cost is not the primary goal, designers of exoskeletal technologies must be able to offset the detrimental impact of device mass and inertia. Users may be willing to sacrifice modest increases in energy expenditure for devices that offer increased protection or capabilities, but even maintaining metabolic cost requirements close to normal physiological levels is often a difficult technological challenge. Additional mass added to the body results in increased biological joint work and metabolic rate. During level walking, Browning et al. [7] showed that the metabolic increase associated with adding mass to the foot is more than four times greater than the same mass attached to the waist. Lower-extremity exoskeletons counteract the effects of device mass through assistive exoskeletal forces and moments applied to the body. The energy needed for locomotion is shared between the walking human and the worn exoskeleton with the goal of maintaining or even lowering the human energetic contribution as compared to normal physiological levels required when the device is not worn.

Exoskeletons can be classified into two categories: passive and active. Passive exoskeletons are often lightweight, but due to their lack of power supply and electronics, their controllability is limited [4, 8]. Active exoskeletons generally implement electronic control systems that can modulate exoskeletal behaviors for different conditions [9, 10], including the adjustment of positive power to the user [1–3, 11, 12]. Furthermore, exoskeletons can be classified as either tethered or autonomous. Tethered exoskeletons require a connection (e.g., pressurized lines, electrical wires) to an external mass not worn on the body, typically an energy source or control hardware [1, 10]. Unlike tethered exoskeletons, the entire system of an autonomous exoskeleton is worn by the user [3, 8, 13, 14], and thus, autonomous exoskeletons are typically not limited to a laboratory setting.

Passive exoskeletons have been shown to reduce the metabolic cost of cyclic motions using lightweight designs that store and release substantial amounts of strain energy. In the study of Grabowski & Herr [8], a lightweight, passive exoskeleton was shown to significantly reduce the metabolic cost of hopping by an average of 24 %. The hopping exoskeleton used leg-parallel fiberglass leaf springs between the user’s feet and hips to store and release energy. During each hop, approximately half of the required eccentric and concentric leg work was provided by the exoskeleton’s leg springs, likely reducing the metabolic cost by reducing the mechanical muscle-tendon work. The large elastic recoil and low device mass both contributed to the success of the hopping exoskeleton. The knee joint in human hopping can be approximated as a torsional spring, exerting minimal torque during full knee extension with torque increasing with increasing knee flexion angle. In the study of Farris et al. [15], a passive elastic ankle exoskeleton was shown to significantly reduce the cost of hopping by 13 %. Wearing the elastic exoskeleton also resulted in both a reduction of soleus activation and force generation, but a reduction in positive fascicle power was not observed. The biomechanical profile of hopping enables simple and lightweight exoskeletal designs that can passively store elastic energy during knee flexion and ankle dorsiflexion and then release that stored energy during knee extension and ankle plantar flexion.

Recently, a passive ankle exoskeleton was shown to reduce the metabolic cost of walking at 1.25 m/s by 7.2 % [4]. The exoskeleton used a steel coil spring and a mechanical clutch connected to carbon fiber shank and foot frames customized for each participant. Wearing a moderately stiff spring resulted in a reduction of the biological component of average ankle moment, positive ankle power, and soleus muscle activation during the stance phase of walking. Unlike the ankle, biological hip and knee powers did not appear to change substantially while wearing the exoskeleton.

Practical applications such as permanent assistive devices and augmentative exoskeletons for recreation or military purposes require autonomous devices where the human wearer carries their own source of energy and control. Such active autonomous leg exoskeletons have been designed with the aim of augmenting running and walking. Elliot et al. [9, 16] developed an autonomous active exoskeleton for running using the observation that the biological knee can be approximated as a spring during the stance phase of slow to moderate-speed running. Similar to the hopping exoskeleton of Grabowski & Herr [8], the running exoskeleton also used fiberglass leaf springs on the distal and proximal sides of the knee, but the static knee joint of the hopping exoskeleton was replaced with a controllable, high-torque and lightweight clutch. The articulated knee joint allowed the leg to freely flex during the swing phase and then locked the joint during stance to enable storage and release of strain energy in the leg-parallel leaf springs. Upon evaluation, the device was not shown to reduce the metabolic cost of running. The authors argued [16] that the lack of metabolic augmentation was due to the negative impact of device added mass, poor human-device mechanical energy transfer, and insufficient energy return from the parallel leg springs.

In order to mitigate the effects of artificial joint alignment and device inertia, Asbeck et al. developed a soft exosuit [12, 14]. Bowden cable actuators were mounted outside of a backpack near the wearer’s pelvis to reduce the added distal mass of the exoskeleton. The exosuit used textiles and the actively controlled Bowden cables to apply tensile forces in parallel with the lower-extremity muscles. The Bowden cable was routed along the anterior surface of the thigh, passing laterally across the knee axis of rotation and then terminating at the heel. This mechanical routing allowed the worn exoskeleton to simultaneously apply hip flexion and ankle plantar flexion moments. During level ground walking, the powered exosuit was shown to reduce the metabolic cost of walking when compared to the powered-off exosuit [12]. However, it has not been shown to reduce the metabolic cost of walking when compared to the no exosuit condition, possibly a consequence of the 10.1 kg system mass.

One effective strategy for reducing the mass of a worn active exoskeleton is to move the energy source and control hardware off the body [1, 10]. For example, the worn mass of pneumatic exoskeletons can be reduced by tethering the device to off-board compressed air and control valves. A tethered pneumatic ankle exoskeleton was shown to reduce the metabolic cost of level-ground walking by 6 % when compared to normal walking [1]. Tethered exoskeletons provide a means to study the effects of active exoskeletons with a high specific power, but their range is limited to within the laboratory.

An autonomous active ankle exoskeleton was shown to significantly reduce the metabolic cost of walking by 10 % [2], and the cost of loaded walking by 8 % [3]. The device used a lightweight winch actuator to apply large amounts of positive power to the ankle during the powered plantar flexion phase of gait. The unidirectional actuator prevented the device from impeding the user during the swing phase and simplified the control strategy. Although this autonomous exoskeleton has been shown to reduce walking metabolism, the device’s impact on gait biomechanics has not been adequately addressed. As authors Galle et al. [11] have suggested, full kinematic and kinetic data are needed to understand the biomechanical impact of an metabolically-augmentative exoskeleton.

The purpose of this study is to investigate the biomechanical impact of wearing an active autonomous exoskeleton that has been previously shown to reduce the metabolic cost of human walking [2]. The intent of this research is to understand how the body cooperates with an augmentative ankle device in order to inform future exoskeletal control and hardware design. We hypothesize that an ankle exoskeleton designed to assist powered plantar flexion will reduce the metabolic cost of walking by primarily reducing the mean positive ankle power provided by the body. To evaluate this hypothesis, six study participants walked on a level treadmill at 1.4 m/s under three conditions: exoskeleton not worn, exoskeleton worn in a powered-on state, and exoskeleton worn in a powered-off state. Metabolic rates were measured with a portable pulmonary gas exchange unit, body marker positions with a motion capture system, and ground reaction forces with a force-plate instrumented treadmill. An inverse dynamics calculation was then performed using the motion capture and force plate data to estimate exoskeletal ankle mechanics and biological ankle, knee and hip kinetics for each walking condition. The resulting energetics and biomechanics data are presented along with implications for future exoskeleton design and control.

## Methods

### Exoskeleton

**Fig. 1:**
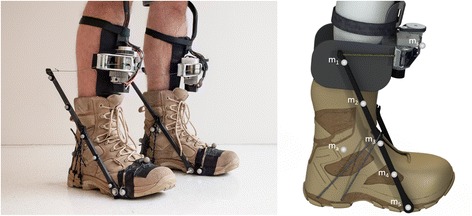
Active autonomous ankle exoskeleton. The active autonomous ankle exoskeleton used a winch actuator on the shin to actuate the proximal ends of fiberglass struts attached to the boot. The winch actuator implemented a brushless DC motor and pulley transmission to wind a high strength cord. The motor controllers and batteries were worn around the chest and waist. Reflective markers on the strut were used to measure the deflection of the struts and the applied exoskeletal torque

The active autonomous exoskeleton used in this study was previously presented in [2]. The exoskeleton was designed to minimize distal mass while maximizing mechanical power output, efficiency and comfort. The exoskeleton design relied on the support of the human ankle to supply structure to the mechanism. The lack of an artificial joint parallel to the human ankle joint both reduced the device mass and eliminated the need for joint alignment or the unwanted forces that might arise from joint misalignment. In order to increase comfort and efficiency, forces were only applied to the shank and heel in (ideally) the direction normal to the skin’s surface. The minimal amount of soft tissue on the anterior aspect of the shank provided an efficient area to transfer load between the exoskeleton and human body. Both the heel and shank are also capable of comfortably withstanding substantial normal forces. The bilateral exoskeleton consisted of pairs of fiberglass struts attached to each boot, winch actuators on each shank (Fig. 1), and a vest containing the batteries and motor controllers.

Two fiberglass struts were attached to each boot to create a large moment arm for the winch actuator. In order to attach the struts, three holes, perpendicular to the sagittal plane, were drilled into the sole of each boot: one under the metatarsophalangeal joints and two under the heel. Each strut measured approximately 380 mm long, 7 mm wide, and 5–20 mm thick. The shape of the strut was designed to minimize mass by enforcing near-constant surface strain on the anterior and posterior faces, and avoiding lateral torsional buckling during actuation. The distal ends of the struts were pinned to the boots on the medial and lateral aspects of the metatarsophalangeal joints. Approximately 190 mm from the distal end, the struts were attached to the heel of the boot with an inextensible cord; the angle between the bottom of the foot and struts was approximately 0.9 radians, creating a moment arm of 230 mm. These strut dimensions were held constant across all subject. The entire exoskeletal structure attached to each boot was 190 g. The same boot model (model: T180 X-Force, Wellco Enterprises, Morristown, TN) was used for all walking conditions, and had a mass of 750 g.

The autonomous exoskeleton used a pair of winch actuators (one on each shin) to exert a plantar flexion torque about the ankle. A 200 W brushless motor (model: 305015, Maxon Motor, Sachseln, CH) actuated an 8 mm diameter spool through a single stage pulley transmission with a speed reduction of 44:14. The spool wound up a high strength ultra-high-molecular-weight polyethylene cord (1 mm diameter) to exert a force on the proximal ends of the fiberglass struts. An aluminum housing constrained the motor, spool and idler pulleys. Although the housing acted as a heat sink for the motor, additional aluminum finned heat sinks were attached to the motors, reducing the thermal resistance between the motor housing and the environment. Custom 3D-printed shin guards were used to distribute the force from the winch actuator and support calf guards to protect the medial and lateral sides of the leg from the proximal ends of the struts. The shin guard also supported a single axis (normal to the sagittal plane) gyroscope for gait phase detection. The winch actuator had a mass of 870 g per leg.

In order to reduce distal mass, the batteries and motor controllers were attached to a vest. The angular position of each motor was measured by a 500 count quadrature incremental optical encoder (model: HEDL 5540, Avago Technologies, San Jose, CA). The actuators were controlled by brushless motor controllers (model: SBL1360, Roboteq, Scottsdale, AZ) that supported sensory acquisition, power electronics and the onboard microprocessor. Two 24 V, 2.5 Ah, lithium-polymer batteries in series powered the entire system. The mass of the batteries and motor controllers was 1480 g, resulting in a total device mass of 3600 g.

Due to the unidirectional nature of the winch actuator, a finite state machine was used to apply assistance during stance, and apply zero force during swing. The gyroscope located on the shin was used to determine a swing event. A swing event was detected if the shank underwent a prolonged period (over 220 ms) of protraction. A heel strike was then estimated as occurring at the moment of retraction, when the shank angular velocity crossed through zero. A heel strike event initiated cord tension and the power timer. The actuator was controlled to apply a slight plantar flexion torque to maintain cord tension until the power timer expired (400–500 ms). After the power timer expired, a parabolic voltage profile (with a peak of approximately 25 V) was applied to the ankle joint over 150 ms. The end of power assistance initiated the swing phase, during which the actuator quickly released the cord to not impede the user during dorsiflexion. The device remained in a swing phase until the next heel strike was detected. After each gait cycle (heel strike to heel strike), an adaptive controller was used to adjust the power timer. The power timer was automatically and incrementally adjusted during the trial so that the peak actuator power would occur at 53 % gait cycle. The timing was selected to align with literature values of ankle power timing [17].

### Exoskeletal torque measurement

In order to simplify the exoskeletal design, reduce device mass, and simplify data acquisition, the torque applied to the ankle by the exoskeleton was measured by the motion capture system. Five 6 mm reflective markers were placed on each lateral fiberglass strut (Fig. 1). The markers are denoted by *m*_*i*_, where *i* =1 is the most proximal marker and *i* = 5 is the most distal marker. While recording the position of the body markers, the motion capture system simultaneously recorded the position of the strut markers. Due to the flexibility of the struts, the changing shape of the strut could be measured by observing the relative positions of the strut markers. The shape of the strut was measured by projecting the five marker positions, *m*_*1–5*_, onto the sagittal plane. The collection of two-dimensional points were then rotated so m1 and m5 would lie on the *x*-axis. A quadratic function, *G*(*x*) = *g*_1_*x*^2^ + *g*_2_*x* + *g*_3_, was fitted to the five points using the method of least squares. The component of the string force perpendicular to the strut, *F*_*⊥*_, was estimated as being proportional to the second derivative of the fitted quadratic function,

1 $$ {F}_{\perp}\propto \frac{d^2G(x)}{d{x}^2}\propto 2{g}_1. $$

The unit direction parallel to the strut, *û*_∥_, was calculated as the normalized difference between the most proximal and distal marker,

2 $$ {\widehat{u}}_{\parallel }=\frac{{\overrightarrow{m}}_1-{\overrightarrow{m}}_5}{\left|\right|{\overrightarrow{m}}_1-{\overrightarrow{m}}_5\left|\right|{}^{,}} $$

and the unit direction perpendicular to the strut, *û*_⊥_, is simply a rotation of *û*_∥_,

3 $$ {\widehat{u}}_{\perp }=\left[\begin{array}{cc}\hfill 0\hfill & \hfill 1\hfill \\ {}\hfill -1\hfill & \hfill 0\hfill \end{array}\right]{\widehat{u}}_{\parallel }. $$

The proximal tip of the strut, *m*_*t*_, was then estimated as being 30 mm beyond the most proximal marker,

4 $$ {\overrightarrow{m}}_t={\overrightarrow{m}}_5+0.03\ {\widehat{u}}_{\parallel }. $$

The unit direction of the string force, *û*_*string*_, was estimated as being a tension force in the unit direction defined by the  sagittal plane projection of the anterior shank marker *m*_*s*_, and the proximal strut tip

5 $$ {\widehat{u}}_{string}=\frac{m_s-{m}_t}{\left|\right|{m}_s-{m}_t\left|\right|{}^{.}} $$

With the proportional magnitude and direction of the perpendicular strut force, and the string direction, the string force, *F*_*s*_, was estimated as

6 $$ {F}_s=a\frac{g_1}{{\widehat{u}}_{\perp}\cdot {\widehat{u}}_{string}}+b, $$

where a and b are experimentally determined constants. The torque applied by the exoskeleton, *τ*_*exo*_, was calculated as the cross product of the moment arm and the estimated string force,

7 $$ {\tau}_{exo}=\left[\begin{array}{c}\hfill {\overrightarrow{m}}_t-{\overrightarrow{m}}_a\hfill \\ {}\hfill 0\hfill \end{array}\right]\times \left[\begin{array}{c}\hfill {F}_s\ {\widehat{u}}_{string}\hfill \\ {}\hfill 0\hfill \end{array}\right], $$

where *m*_*a*_ is the sagittal plane projection of the lateral ankle joint marker. This method enabled a measurement of exoskeletal torque that was synchronized with the human biomechanics at the minimal mass cost of five reflective markers per leg.

The constants *a* and *b* were experimentally determined in a calibration experiment. An inline force sensor (model: LRF350, Futek Advanced Sensor Technology Inc., Irvine, CA), with its own reflective marker, was used to apply various load conditions on the proximal tip of the struts. The magnitude of force, direction of force, and frequency of force were varied over approximately 60 s. The force sensor and three-dimensional positions of the strut markers and sensor marker were simultaneously measured. Using the methods described above, the applied exoskeletal torque was estimated, and the force sensor was used to directly measured the torque, *τ*_*sensor*_, which was calculated as the cross product of the moment arm and the measured force, *F*_*sensor*_,

8 $$ {\tau}_{sensor}=\left[\begin{array}{c}\hfill {\overrightarrow{m}}_t-{\overrightarrow{m}}_a\hfill \\ {}\hfill 0\hfill \end{array}\right]\times \left[\begin{array}{c}\hfill {F}_{sensor}\ {\widehat{u}}_{string}\hfill \\ {}\hfill 0\hfill \end{array}\right]. $$

In the calculation of *û*_*string*_, the marker on the force sensor was used in place of the shank marker, *m*_*s*_. The method of least squares was used to determine the constants *a* and *b* from Eq. 6 that minimized the mean square error between *τ*_*exo*_ and *τ*_*sensor*_.

### Experimental protocol

The biomechanical effects of the autonomous powered exoskeleton were experimentally determined using six study participants (6 male; 89 ± 8 kg body mass, 183 ± 6 cm in stature; 27 ± 4 years old; mean ± standard deviation). All subjects were healthy and exhibited no gait abnormalities. This study was approved by the MIT Committee on the Use of Humans as Experimental Subjects, and all of the participants gave their informed consent after the nature and possible consequences of the experiment were explained. The participants were asked to perform one standing trial and then four walking trials. Walking trials were performed on a treadmill at a speed of 1.4 m/s, approximately the average adult walking speed [18]. Participants were first asked to walk 10 min without the exoskeleton, then 20 min with the active exoskeleton, 20 min with the exoskeleton in a powered-off state, and finally, 10 min without the exoskeleton. Walking experiments began and ended without the exoskeleton to account for natural variations in metabolism.

### Data analysis and processing

The net metabolic cost of walking during each condition was estimated by measuring the exchange of oxygen and carbon dioxide during quiet standing and walking. During the standing and walking trials, participants wore a portable pulmonary gas exchange measurement instrument (model: K4b^2^, COSMED, Rome, IT). The measured exchange rates of oxygen and carbon dioxide along with the equation developed by Brockway et al. were used to estimate the metabolic rate [19]. The metabolic rate for a given trial was determined by taking the mean rate over the last 5 min of the trial. The measurements made during the two walking trials without the exoskeleton were averaged to determine a single metabolic rate without the exoskeleton. The metabolic rate measured while standing was subtracted from the metabolic rates of walking in order to obtain the net metabolic cost of walking.

Kinematic and kinetic data were simultaneously collected with the metabolic measurements. To measure kinematic and kinetic data, participants walked on a \split-belt instrumented treadmill (Bertec, Columbus, OH) within a motion capture volume. Each tread independently measured shear and vertical forces at a sampling rate of 1000 Hz. Reflective markers (15 mm diameter) were placed on the participant’s body at 46 locations, and their three-dimensional locations were measured at a sampling rate of 100 Hz by 12 infrared cameras (model: T40s, Vicon Motion Systems Ltd, Oxford, UK). The locations of these markers followed the Helen Hayes marker model, and were chosen to track joint motion. Markers were not placed on the posterior side of the shank due to exoskeleton interference. Two pairs of boots (same model number) were used during the experiments; one pair while walking with the powered and unpowered exoskeleton, and one pair while walking without the exoskeleton. Features on the boots were used to duplicate marker positions as closely as possible. Five additional markers (6 mm) were placed on each lateral fiberglass strut. These markers were used to measure the applied exoskeletal torque and are discussed in the previous section.

Various post-processing steps were performed to produce average kinematic and kinetic profiles for each subject. The maker position data coupled with SIMM software (Software for Musculoskeletal Modeling, Musculographics Inc., Evanston, IL) were used to compute joint angles. These kinematic data were then combined with the force plate measurements and analyzed with the SIMM Dynamics Pipeline to compute joint moment profiles. A fourth order Butterworth filter with a 6 Hz cutoff frequency was used to filter the kinematic data [20], and a fourth order Butterworth filter with a 25 Hz cutoff frequency was used to filter the force plate measurements. Although SIMM provides a complete set of profiles in three-dimensional space, only sagittal plane motion was considered in this study.

Joint powers were calculated as the product of joint velocity and joint torque, where positive power is defined as joint or exoskeleton torque in the same direction as joint velocity. Mean positive power was defined as the time integral of positive power divided by the gait cycle time, and mean negative power was defined as the time integral of negative power divided by the gait cycle time. During the active exoskeleton condition, total ankle (biological + exoskeletal) moment and power were calculated by inverse dynamics. The biological contribution was calculated by subtracting the exoskeletal contribution from the total ankle value.

The kinematic and kinetic profiles were calculated for the last 30 s of each walking trial. A heel strike was recorded if the vertical force on the tread exceeded 0.5 N after a prolonged period (greater than 200 ms) of no force, corresponding with a swing phase. Each 30 s trial was divided into individual gait cycles that began and ended with heel strike of the same foot, and then normalized in time by percent gait cycle. Moments and powers were also normalized by the subject’s body mass. Gait cycles were discarded when the participant’s foot simultaneously contacted both force plates. Mean joint profiles (e.g., angle, moment, power) were calculated for each leg during a trial. The right and left leg profiles were then averaged together to calculate a subject’s mean profile for a given trial. Furthermore, the two without exoskeleton trials were averaged together to create a single set of without exoskeleton profiles. The mean profiles of each subject were then used to calculate the intersubject mean profile and standard error at each point in the gait cycle. Individual scalar outcomes (e.g., mean powers, peak angles, peak moments) were calculated from the mean profile of each subject. The individual scalar outcomes were then used to calculate the intersubject mean and standard error.

### Statistical analysis

We compared the metabolic cost of walking and scalar biomechanical measures across the three conditions: walking without an exoskeleton, walking with the active exoskeleton and walking with the powered-off exoskeleton. We first performed a repeated-measures ANOVA with the level of significance set at 0.05. The exoskeleton condition was treated as a fixed factor and the subject was treated as a random factor. When we found a significant effect, post-hoc pairwise comparisons were made with a Tukey honestly significant difference test, with the level of significance set at 0.05.

## Results

### Torque measurement calibration

**Fig. 2:**
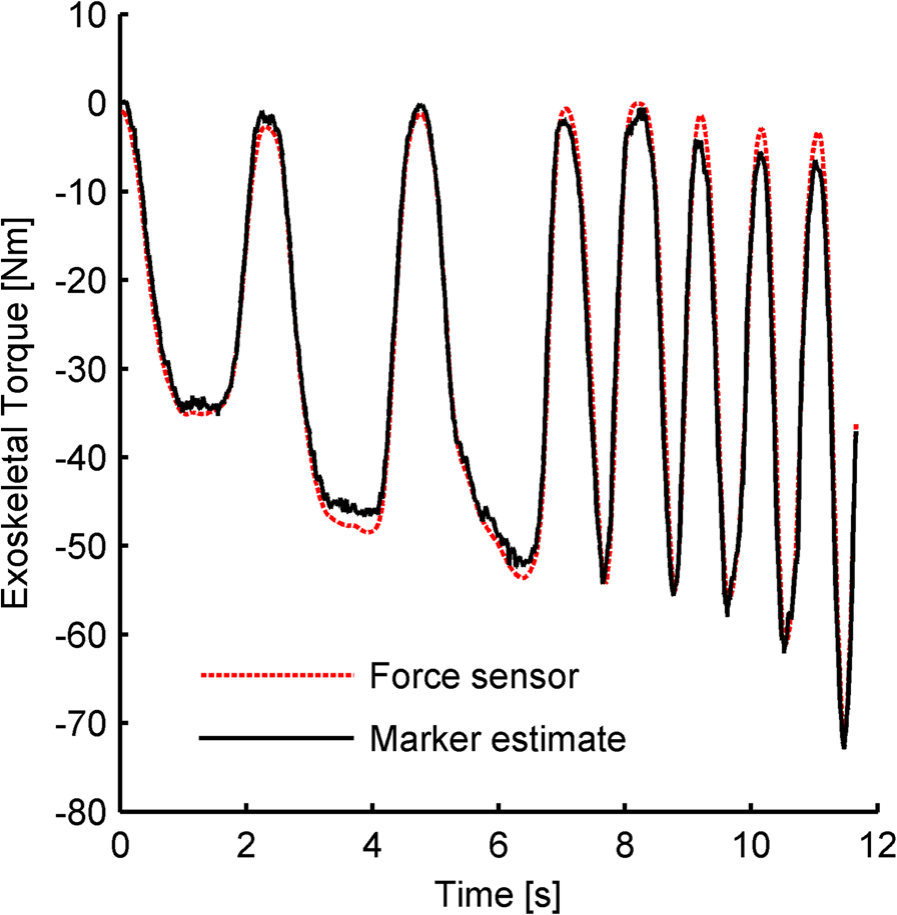
Strut torque characterization. The exoskeletal torque measured by a force sensor and motion capture system were compared to the exoskeletal torque predicted by the deflection of the struts. Using reflective markers on the strut enabled a simple, synchronous method for measuring exoskeletal torque without adding substantial mass. The system was calibrated at various amplitudes and frequencies, similar to those experienced during walking. Only a portion of the calibration data are shown in the figure

The relationship between the fiberglass strut’s shape and the applied exoskeletal torque was experimentally determined with a digital force sensor. The applied exoskeletal torque was varied between 0 and 70 Nm, at frequencies between approximately 0 and 3 Hz. These input profiles were chosen to be similar to the conditions experienced during walking, since the force could not be directly measured during walking. After performing calibration experiments on both the right boot struts and the left boot struts, the constants *a* and *b* from Eq. 6 were determined to be

9 $$ {F}_s=897\frac{g_1}{{\widehat{u}}_{\perp}\cdot {\widehat{u}}_{string}}-36, $$

with an R^2^ value of 0.97. The negative y-intercept is a result of the relaxed strut markers not being perfectly collinear and thus measuring a non-zero second derivative. Using Eqs. 7, 8 and 9, the exoskeletal torque estimated by the markers was compared to the exoskeletal torque measured by the force sensor (Fig. 2). The root mean square error of the exoskeletal torque was calculated to be 3.2 Nm, or approximately 5 % of the peak measured torque.

### Metabolic cost and joint mechanics

The active ankle exoskeleton significantly reduced the metabolic cost of walking. Net metabolic cost of walking with the active exoskeleton (3.28 ± 0.10 W/kg) was an 11 ± 4 % (*p* = 0.019) reduction compared to the cost of walking without the exoskeleton (3.71 ± 0.14 W/kg), and a 14 ± 3 % (*p* < 0.01) reduction compared to walking with the powered-off exoskeleton (3.82 ± 0.13 W/kg). To achieve this observed metabolic reduction, the exoskeleton provided a mean positive power of 0.105 ± 0.008 W/kg to the ankle joint, and a net power of 0.074 ± 0.006 W/kg. Here all measurements are given as mean ± standard error.

**Fig. 3:**
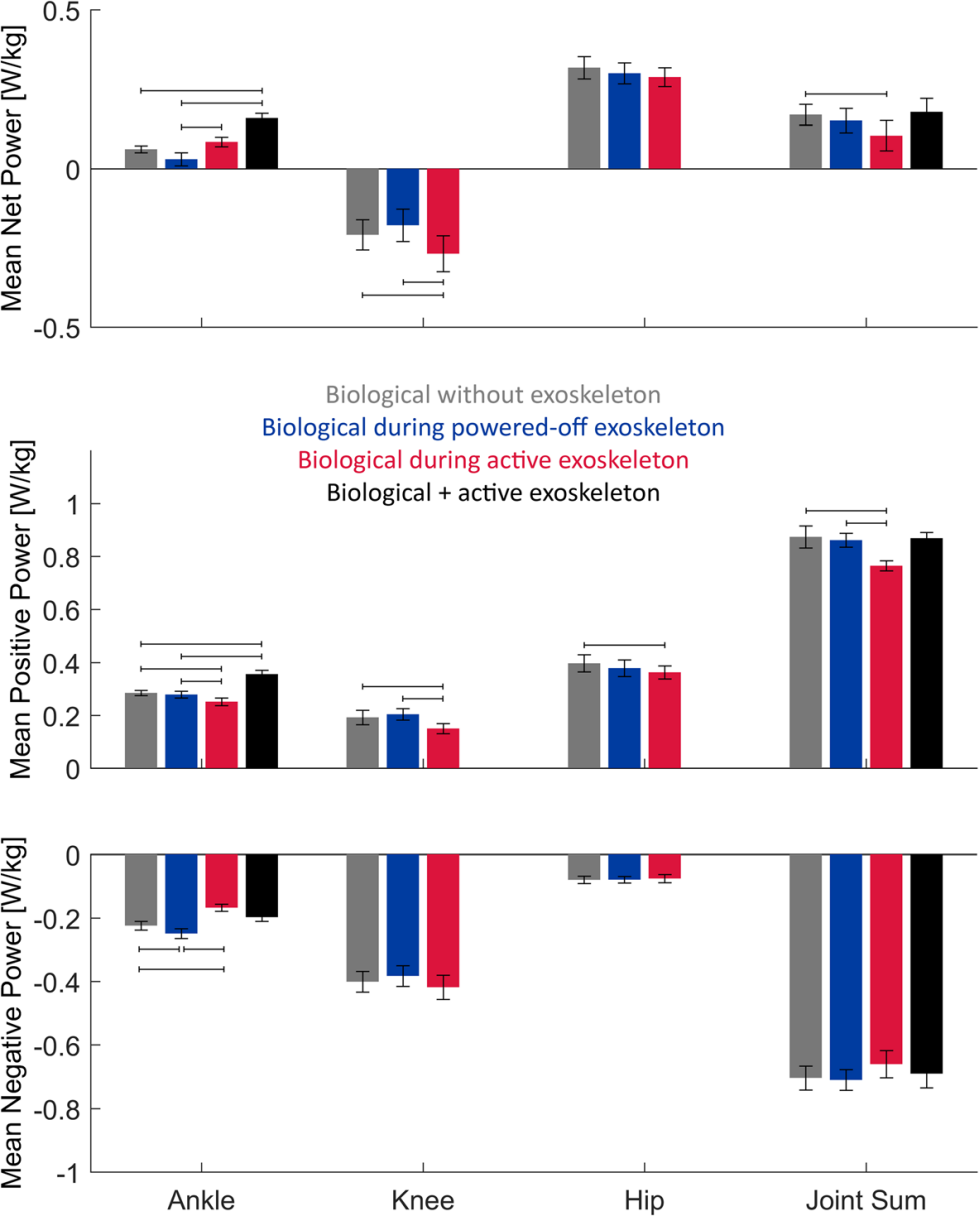
Exoskeletal effects on mean joint powers. The mean net powers, mean positive powers, and mean negative powers of the biological ankle, knee, hip and sum of all three joints are shown while wearing no exoskeleton (*grey*), the powered-off exoskeleton (*blue*), and the active exoskeleton (*red*). The black bars also include the mechanical exoskeletal power. Vertical error bars represent the standard error means, and horizontal brackets denote conditions that are significantly different (*p* < 0.05)

In addition to reducing walking energetics, the device altered walking biomechanics. For the three experimental conditions, the  mean mechanical powers of the biological ankle, knee and hip joints are shown in Fig. 3. Although the active exoskeleton did significantly reduce the mean positive power of the biological ankle joint, it also significantly reduced the mean positive power of both the knee and hip joints. Compared to walking without the exoskeleton, wearing the active exoskeleton significantly reduced mean positive power of the biological ankle by 0.033 ± 0.006 W/kg (*p* < 0.01), the knee by 0.042 ± 0.015 W/kg (*p* = 0.02), and the hip by 0.034 ± 0.009 W/kg (*p* < 0.01). Also, compared to walking without the exoskeleton, wearing the exoskeleton in a powered-off state was not shown to significantly alter the mean positive powers at the ankle, knee or hip.

The sum of biological joint powers and exoskeletal power (during the active condition) were also compared across experimental conditions. The results are shown in Fig. 3. Wearing the active exoskeleton significantly reduced the sum of biological joint mean positive powers by 0.109 ± 0.025 W/kg (*p* < 0.01) when compared to the no exoskeleton condition, but wearing the powered-off exoskeleton did not have a significant effect (*p* = 0.838). When the mean positive power of the exoskeleton was added to the sum of biological mean positive powers during the active exoskeleton condition, exoskeletal condition was not observed to have a significant effect (ANOVA, *p* = 0.833). These results show that the active exoskeleton reduced the mean positive power of the biological muscle-tendon system, but the mean positive power of the total system (biological + exoskeleton) remained unchanged.

**Fig. 4:**
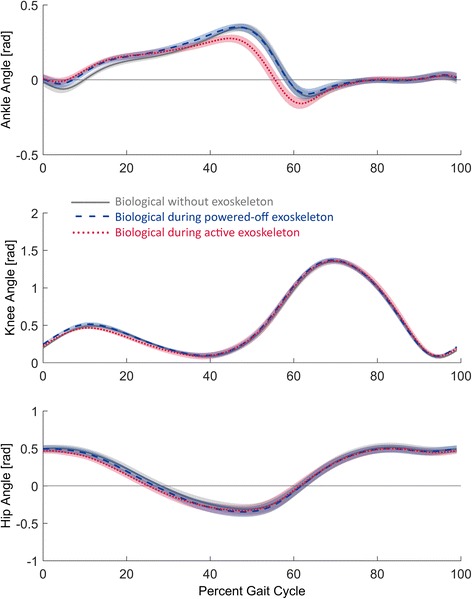
Joint angle profiles. The angle trajectories of the ankle, knee and hip are shown over a gait cycle that begins and ends with heel strike of the same leg. Increasing angles represent dorsiflexion at the ankle, flexion at the knee, and flexion at the hip. The trajectories are intersubject means. The grey solid lines represent the no exoskeleton condition; dark blue dashes represent the powered-off exoskeleton condition, and the red dots represent the active exoskeleton condition. The standard error means are shown with light shading of the same color

**Fig. 5:**
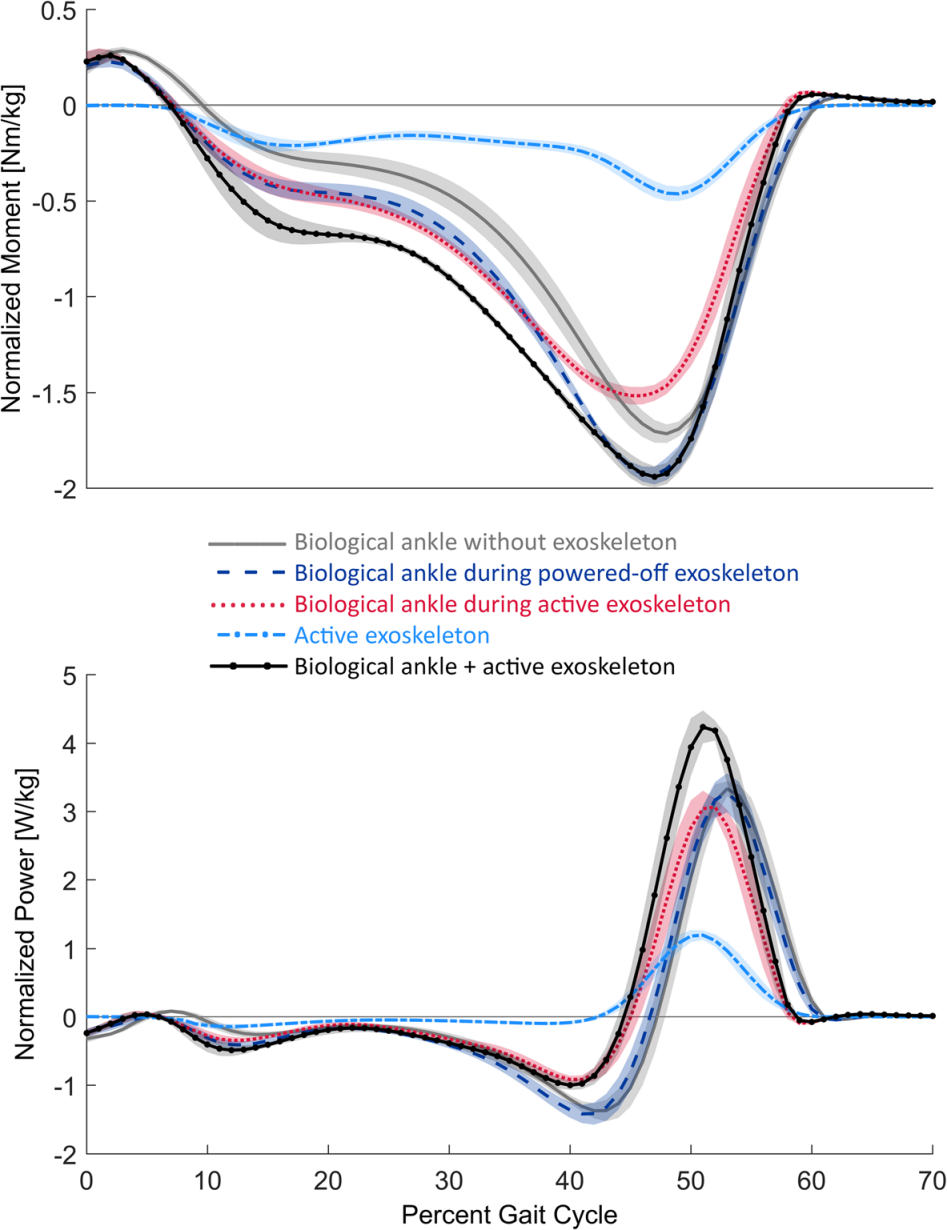
Ankle moment and power profiles. The ankle moment profiles during the first 70 % of the gait cycle are depicted on the top graph, and the ankle power profiles are depicted on the bottom. Positive moment values denote ankle dorsiflexion. The figure compares the biological ankle during the no exoskeleton condition (*grey solid line*), the powered-off exoskeleton condition (*dark blue dashes*), and the active exoskeleton condition (*red dots*). The exoskeleton moment and power during the active condition are shown with light blue dashes and dots. The sum of the biological ankle and exoskeleton during the active condition are shown with a solid black line with dots. The standard error means are shown with light shading of the same color

**Fig. 6:**
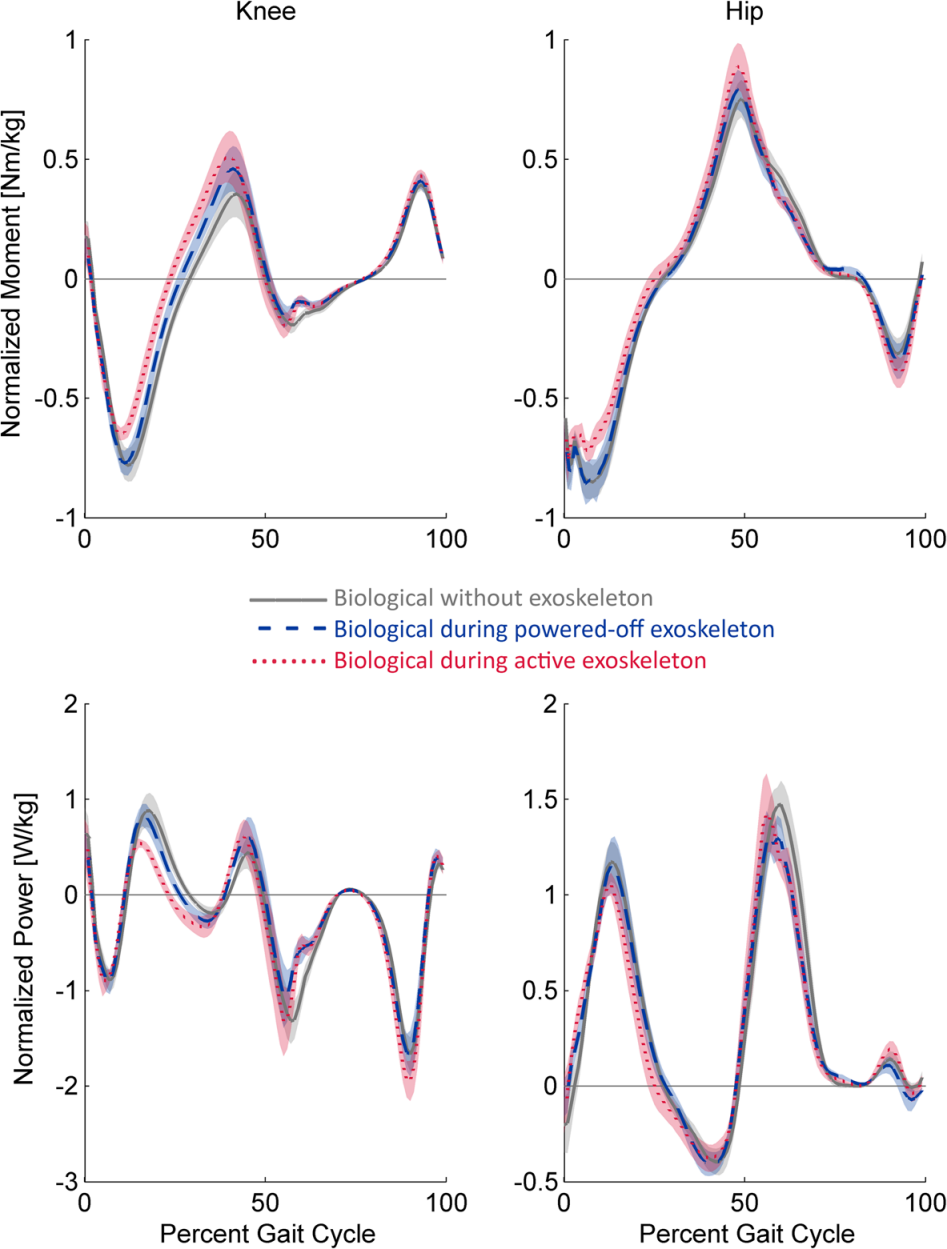
Knee and hip moment and power profiles. The knee and hip moment and power profiles are compared during the three exoskeletal conditions. Positive moment values denote flexion at the knee and flexion at the hip. The moment profiles are shown on top and the power profiles are shown on the bottom. The knee profiles are on the left and the hip profiles are on the right. The grey solid lines represent the no exoskeleton condition; dark blue dashes represent the powered-off exoskeleton condition, and the red dots represent the active exoskeleton condition. The standard error means are shown with light shading of the same color

The mechanisms by which the exoskeleton affected the biomechanics were investigated by comparing intersubject mean joint angles, moments and power trajectories during the different walking trials (Figs. 4, 5, and 6). Comparing the kinematic and kinetic trajectories show both when and how the exoskeleton affected the biomechanics of walking.

The kinematic changes caused by the active ankle exoskeleton were limited to the ankle joint (Fig. 4). Wearing the active exoskeleton significantly reduced the magnitude of the peak dorsiflexion angle of the biological ankle by 0.073 ± 0.014 radians (*p* < 0.01) when compared to the no exoskeleton condition, but wearing the powered-off exoskeleton did not result in a significant change. Wearing the active exoskeleton also led to an earlier onset of ankle powered plantar flexion. Significant changes in the biological knee and hip angle trajectories were not observed. A significant change in step period was also not observed.

Along with kinematic changes, the joint moment effects of wearing the exoskeleton were measured (Figs. 5 and 6). Compared to walking without the exoskeleton, wearing the active exoskeleton decreased the magnitude of peak biological ankle powered plantar flexion moment by 0.19 ± 0.03 Nm/kg (*p* < 0.01). Interestingly, the magnitude of the peak total ankle moment (biological + exoskeleton) during the active exoskeleton condition was not significantly different from the powered-off condition (*p* = 0.53), but both the active and powered-off exoskeleton conditions were significantly greater than the no exoskeleton condition by 0.25 ± 0.04 Nm/kg (*p* < 0.01) and 0.20 ± 0.05 Nm/kg (*p* < 0.01) respectively. Compared with the no exoskeleton condition, wearing the active exoskeleton also increased the peak knee flexion moment during late stance by 0.11 ± 0.06 Nm/kg (*p* < 0.01), and the peak hip flexion moment by 0.17 ± 0.05 Nm/kg (*p* < 0.01), but reduced the magnitude of the peak hip extension moment by 0.090 ± 0.04 Nm/kg (*p* = 0.041). The peak knee and hip moments while wearing the powered-off exoskeleton were not observed to be significantly different from the no exoskeleton condition.

The ankle angle and torque changes resulted in significant changes of the ankle power profile (Fig. 5). Compared to the no exoskeleton condition, the peak biological ankle power was not significantly affected by the active exoskeletal condition (ANOVA, *p* = 0.63), but the magnitude of the peak negative biological ankle power was reduced by 0.48 ± 0.10 W/kg (*p* < 0.01) when wearing the active exoskeleton, and not significantly affected when wearing the powered-off exoskeleton. Similar to the peak total ankle moment, the peak total ankle power (biological ankle + exoskeleton) was significantly increased by 1.02 ± 0.14 W/kg (*p* < 0.01) while wearing the active exoskeleton, but the magnitude of the peak negative total ankle power was similar to the biological ankle. While wearing the active exoskeleton both the biological ankle and total ankle power profiles became positive and peaked earlier than the powered-off and no exoskeleton conditions.

## Discussion

Autonomous leg exoskeletons have now been shown to reduce metabolic demand in level ground walking, but the biomechanical mechanisms underlying such reductions have not been elucidated. In this study, we hypothesize that an ankle exoskeleton designed to assist powered plantar flexion will reduce the metabolic cost of walking by primarily reducing the mean positive ankle power provided by the body. The results of this study do not support the hypothesis. In addition to reducing the mean positive power of the biological ankle, the ankle exoskeleton substantially reduced both the biological knee and hip mean positive powers. The reduction of mean positive power at all three biological joints suggest that the powered exoskeleton was not only reducing the work done by muscles local to the ankle, but altering the gait to reduce the work done by the larger, more proximal muscles spanning the knee and hip.

The fact that the active ankle exoskeleton impacted muscle function spanning the knee joint is not unexpected given the results of Galle et al. [11]. These researchers measured muscle EMG on study participants walking uphill wearing an active ankle exoskeleton designed to assist powered plantar flexion [11]. They showed that the EMG RMS amplitude was significantly reduced in musculature spanning the knee, namely the vastus lateralis and biceps femoris. The electromyography of these knee muscles was reduced during early stance for the experimental condition of the ankle exoskeleton powered-on, as compared to the powered-off condition.

### Mechanical energetics of the biological joints plus exoskeletal system

Although the principle the body employs to distribute exoskeletal power across the human musculoskeletal system is unknown, wearing the active exoskeleton did not significantly change the system (ankle + knee + hip + exoskeleton) mean power. When comparing the system of the ankle, knee and hip biological leg joints plus the powered ankle exoskeleton to only these biological leg joints while not wearing the exoskeleton, there was no significant difference found in the system mean net power, mean positive power, or mean negative power. In other words, the positive and negative mean mechanical power provided by the exoskeleton replaced positive and negative mean mechanical power at the biological joints.

### Biological plus exoskeletal ankle joint augmentation

The results of this study show that the impact of the active ankle exoskeleton was not to simply replace biological ankle function in walking, but rather to augment the total (biological + exoskeletal) ankle moment and power. While wearing the exoskeleton, the total ankle moment and power is defined as the sum of the contributions from the biological ankle and the exoskeleton. If the exoskeleton were to simply replace the biological ankle function, one would expect the peak total ankle power and the magnitude of the peak total ankle moment to not change compared to the no exoskeleton condition. However, wearing the active exoskeleton caused both the peak total ankle power and the magnitude of the peak total ankle moment to increase during stance. A significant difference in the peak biological ankle power was not detected during the different conditions.

These results are in contrast with the results of Collins et al. [4]. They showed that the peak total ankle power decreased while wearing a passive ankle exoskeleton, and the magnitude of the peak total ankle moment was unaffected by wearing the device. Their passive ankle exoskeleton also reduced the peak biological ankle power. Although both the device of Collins et al. [4] and the device evaluated in this study were designed to assist ankle plantar flexion, distinct human adaptations emerged upon testing each exoskeletal system. These distinct adaptations are consistent with the findings of Jackson and Collins [21], who measured the effects of a tethered unilateral ankle exoskeleton during different power and torque conditions. They observed that the peak total ankle power increased when the exoskeleton provided medium and high amounts of net work, but the peak total ankle power decreased when the device provided zero or negative net work. These findings by Jackson and Collins [21] are consistent with the active ankle exoskeleton of this study increasing peak total ankle power and the passive ankle exoskeleton of Collins et al. [4] reducing peak total ankle power.

### Implications for Exoskeleton Design

In this study, we hypothesized that the ankle exoskeleton would predominantly replace ankle function, although the effects of the device are more global and nuanced than had been initially anticipated. If the device only replaced ankle power, then one would expect the upper boundary of augmentation to be complete replacement of the biological ankle torque. However, if only part of the exoskeletal power is used to lower the biological ankle power and a significant amount is also used to lower the knee and hip biological powers, then it may be possible to achieve even greater metabolic augmentation levels by applying power to the exoskeletal ankle that is greater than the natural biomechanical values observed during unassisted locomotion. This idea is also supported by a previous study on non-amputees wearing prosthetic ankles that showed the metabolic rate continued to decrease even after the ankle power was well beyond a natural level [22]. If this phenomenon extends to ankle exoskeletons, many design benefits could be achieved. First, a single ankle joint exoskeleton that aids multiple biological joints is a simpler and likely lighter solution than a multi-joint exoskeleton. Second, the ankle is an ideal leg joint to supply exoskeletal power since the shin, heel and ground can all comfortably withstand large exoskeletal forces. It is more difficult to efficiently and comfortably apply external loads to the thigh and torso where there are greater amounts of soft tissue.

The biomechanical effects of the ankle exoskeleton suggest that the optimal control strategy to minimize metabolic energy consumption may not be to simply emulate the biological ankle behaviour observed during natural unassisted gait. To develop such an optimal control strategy, a full biophysical model of the lower-extremities would prove critically important. Such a model would allow researchers to predict the biomechanical and metabolic effects of externally-applied exoskeletal forces and masses. The model would likely have to capture muscle morphologies, muscle-tendon interactions, neural reflexes and neural adaptations. Indeed, if such a model existed, researchers could determine optimal exoskeletal torque trajectories to minimize walking metabolism. The maximal metabolic reduction possible with such an optimal ankle exoskeleton may be even larger than the metabolic contribution of the biological ankle during normal, unassisted walking.

### Augmentation Factor

The Augmentation Factor (AF) was proposed as a simple model that aims to approximate the metabolic impact of a lower-extremity exoskeleton [3]. The AF balances the metabolic costs associated with the added device mass and net energy removed from the human system, with the beneficial effects of providing the user with positive mechanical power. The results of this study support a fundamental assumption of the Augmentation Factor [3], namely that positive mechanical exoskeletal power is used by the body to replace positive muscle-tendon power. However, a limitation of the AF is that it does not provide insight into the specific joints or muscles that are affected by assistive exoskeletal power.

### Future design improvements

Improvements to the autonomous exoskeleton may result in even larger metabolic reductions. The shifting of the biological ankle power profile suggests that the exoskeleton may be applying power too early. An adjustment in the power timing, or a non-timing based controller, may result in a more economical use of exoskeletal power. It also appears that the amplitude of the applied power could be substantially higher, even exceeding biological levels. A higher transmission ratio or different motor topology should enable additional power. It may also be possible to tune the stiffness of the fiberglass struts to increase the overall efficiency and enable greater power output. Finally, reducing device mass is always a goal and likely to improve performance.

## Conclusion

In this study, an active autonomous ankle exoskeleton reduced the metabolic cost of level ground walking. The biomechanical mechanism for this observed metabolic reduction is not only a mechanical power reduction at the biological ankle, but also an observed reduction at the biological knee and hip, as compared to walking without the exoskeleton. The sharing of exoskeletal power between biological joints suggests that ankle exoskeletons may be capable of greater metabolic reductions than previously thought. The capacity of an ankle exoskeleton to also augment the knee and hip greatly expands the opportunity for practical application.
